# CAPTURING OLDER ADULT PEER MENTORS' EXPERIENCE WITH COMMUNITY-DELIVERED HEARING CARE THROUGH PHOTOVOICE

**DOI:** 10.1093/geroni/igac059.1495

**Published:** 2022-12-20

**Authors:** Carrie Nieman, Audrey Mossman, Julie Yi, Jonathan Suen, Frank Lin, Sarah Szanton, Hae-Ra Han

**Affiliations:** Johns Hopkins University School of Medicine, Baltimore, Maryland, United States; University of Washington School of Medicine, Seattle, Washington, United States; Johns Hopkins University, Baltimore, Maryland, United States; Johns Hopkins University, Baltimore, Maryland, United States; Johns Hopkins Cochlear Center for Hearing and Public Health, Baltimore, Maryland, United States; Johns Hopkins University School of Nursing, Baltimore, Maryland, United States; Johns Hopkins University, Baltimore, Maryland, United States

## Abstract

While age-related hearing loss is common, disparities in care exist by race, ethnicity, and socioeconomic position. HEARS is a hearing care intervention that incorporates over-the-counter hearing technology, partnering older adults with peer mentors via a community health worker (CHW) model to address disparities. Through a randomized controlled trial, 8 older adult peer mentors delivered the HEARS intervention. We aimed to understand the CHWs’ perspectives on their role through photovoice. Participants took photos illustrating the intervention, their role, and hearing health. Semi-structured interviews (n=4) and a focus group were conducted. CHWs responded positively to the intervention and were satisfied with their role. A notable theme was the value of serving as a peer mentor. Participants valued the opportunity for generativity, learning as older adults, and associated social benefits. Our findings demonstrate an opportunity to engage older adults in the evaluation process, expanding access to hearing care, and in peer mentorship.

